# Diastereoselective Mannich reactions of pseudo-*C*_2_-symmetric glutarimide with activated imines

**DOI:** 10.3762/bjoc.13.244

**Published:** 2017-11-21

**Authors:** Tatsuya Ishikawa, Tomoko Kawasaki-Takasuka, Toshio Kubota, Takashi Yamazaki

**Affiliations:** 1 Division of Applied Chemistry, Institute of Engineering, Tokyo University of Agriculture and Technology, 2-24-16 Nakamachi, Koganei 184-8588, Japan; 2 Department of Biomolecular Functional Engineering, Ibaraki University, Nakanarusawa 4-12-1, Hitachi 316-8511, Japan

**Keywords:** chiral oxazolidinones, diastereoselectivity, Mannich reactions, pseudo-*C*_2_ symmetry, trifluoromethyl

## Abstract

As an extension of the boron enolate-based aldol reactions, the oxazolidinone-installed bisimide **1a** from 3-(trifluoromethyl)glutaric acid was employed for Mannich reactions with tosylated imines **2** as electrophiles to successfully obtain the corresponding adducts in a stereoselective manner.

## Introduction

During these decades, we have been keenly interested in the 3-(trifluoromethyl)glutaric acid derivatives and developed a couple of routes to get successful access to such target molecules with a variety of substituents at the 2-position [1–3]. Previously, the oxazolidinone-installed bisimide **1a** was employed for the crossed aldol reactions by the way of boron enolate which allowed the isolation of optically active lactones in good to excellent yields [4–5]. One of the most intriguing features of this protocol is the fact that the enantiomers at the lactone part were readily obtained only by the selection of the tertiary amines employed in the reaction. It is quite apparent that this success is, at least in part, based on its inherent pseudo-*C*_2_ symmetric structure [6–8] which enables the formation of plural stereogenic centers by a single operation. These promising results prompted us to extend this aldol protocol to its relative, Mannich reactions [9–13] whose details are reported in this article.

## Results and Discussion

On the basis of our previous study [4], the chiral glutarimide **1a** was employed as the starting material and optimization of reaction conditions with benzaldehyde-based imines **2** was performed (Table 1). Lithium enolate by the action of LDA to **1a** was found to be ineffective as long as the imines with benzyl (**2aa**) or Boc (**2ab**) as substituents R were employed (Table 1, entries 1 and 2).

**Table 1:** Optimization of reaction conditions.

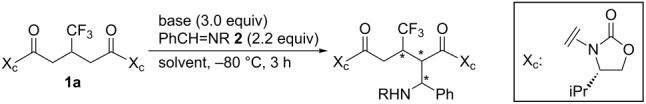					

Entry	Solvent	Base	R	Yield^a^ (%)	DS^b^

1	THF	LDA	PhCH_2_ (**2aa**)	NR	–
2	THF	LDA	Boc (**2ab**)	NR	–
3	THF	LDA	Ts (**2ac**)	85 (**3a**)	69:18:(13)
4	THF	NaHMDS	Ts (**2ac**)	59 (**3a**)	38:43:(19)
5	THF	LiHMDS	Ts (**2ac**)	80 (**3a**)	27:62:(11)
6^c^	DCM	DIPEA	Ts (**2ac**)	NR	–
7^d^	THF	LDA	Ts (**2ac**)	96 [75] (**3a**)	73:17:(10)
8^d,e^	THF	LDA	Ts (**2ac**)	91 (**4a**)	73:21:(6)

^a^All yields were determined by ^19^F NMR and in the bracket isolated yields are given. NR: no reaction. ^b^Diastereoselectivities (DS) were determined by ^19^F NMR and in the parentheses the sum of the other minor stereoisomers is given. ^c^TiCl_4_ (3.0 equiv) was used as a Lewis acid. ^d^The reaction was continued for 6 h. ^e^Substrate **1b** with the phenylglycine-derived oxazolidinone part was employed instead of **1a**.

However, the attachment of the stronger electron-withdrawing 4-toluenesulfonyl (Ts) moiety attained efficient activation of the imine **2ac** to afford the desired Mannich product **3a** in 85% yield as determined by ^19^F NMR (Table 1, entry 3). NaHMDS (Table 1, entry 4) or LiHMDS (Table 1, entry 5) instead of LDA worked properly affording **3a** in a yield of 59 or 80%, respectively. The product **3a** theoretically consists of 8 diastereomers and usually 2 isomers predominated with a couple of minor peaks whose sum is given in parentheses (for example, in the case of entry 3 (Table 1), four small peaks were observed in addition to the major two). It is interesting to note that MHMDS (M: Li or Na) and LDA furnished a different isomer as the major product. We have also prepared the titanium enolate [14], but the reaction did not proceed at all (Table 1, entry 6). As the separation of product **3a** from unreacted substrate **1a** proved to be difficult, the reaction time was extended from 3 h to 6 h. After this time the consumption of **1a** was complete and the yield of **3a** was almost quantitative (Table 1, entry 7). Because the employment of the phenylalanine-based oxazolidinone as the chiral auxiliary under the same conditions gave similar results as the one obtained from valine (Table 1, entries 8 vs 7), we decided the conditions in entry 7 (Table 1) as the best of all tested and investigated the structural scope of imines **2** at the next stage (Table 2).

**Table 2:** Scope and limitation of the present Mannich reactions.

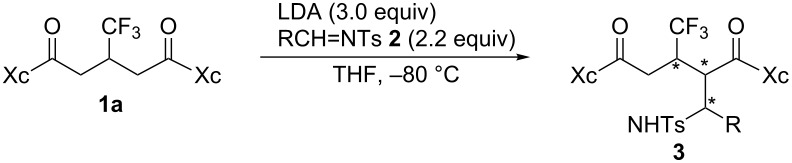						

Entry	Base	R	Time (h)	Yield^a^ (%)	Product	DS^b^

1	LDA	Ph (**2ac**)	3.0	96 [75]	**3a**	73:17:(10)
2	LiHMDS	Ph (**2ac**)	6.0	96[74]	**3a**	27:63:(10)
3	LDA	4-BrC_6_H_4_- (**2bc**)	6.0	93 [91]	**3b**	62:17:(21)
4	LiHMDS	4-BrC_6_H_4_- (**2bc**)	6.0	84 [62]	**3b**	26:61:(13)
5	LDA	4-O_2_NC_6_H_4_- (**2cc**)	6.0	quant [92]	**3c**	65:13:(22)
6	LiHMDS	4-O_2_NC_6_H_4_- (**2cc**)	6.0	64 [45]	**3c**	49:44:(7)
7	LDA	3-FC_6_H_4_- (**2dc**)	6.0	89 [78]	**3d**	81:6:(13)
8	LiHMDS	3-FC_6_H_4_- (**2dc**)	6.0	84 [72]	**3d**	33:51:(16)
9	LDA	2-furyl- (**2ec**)	8.0	95 [55]	**3e**	51:22:(27)
10	LiHMDS	2-furyl- (**2ec**)	6.0	48	**3e**	28:57:(15)
11	LDA	4-MeC_6_H_4_- (**2fc**)	8.0	quant [66]	**3f**	51:16:(33)
12	LiHMDS	4-MeC_6_H_4_- (**2fc**)	6.0	51	**3f**	28:64:(8)
13	LDA	4-MeOC_6_H_4_- (**2gc**)	6.0	86	**3g**	34:31:(35)
14	LiHMDS	4-MeOC_6_H_4_- (**2gc**)	6.0	26	**3g**	38:29:(33)
15	LDA	Et (**2hc**)	6.0	93	**3h**	39:36:(25)
16	LDA	iPr (**2ic**)	6.0	23	**3i**	37:17:(46)

^a^All yields were determined by ^19^F NMR and in the bracket the isolated yields are given. ^b^Diastereoselectivities (DS) were determined by ^19^F NMR for the crude mixture and in the parentheses the sum of the other minor stereoisomers is given.

As mentioned above, LDA and LiHMDS proved to work in a complementary manner in terms of their diastereoselectivity towards products **3**. Therefore, we next tried to use both bases for the reaction with **1a** and the resultant enolate was treated with a variety of tosylimines **2** (Table 2). For better overview entry 1 in Table 2 contains the same data as entry 7 in Table 1. It is assumed that the differences between the spectroscopically determined yields to the isolated ones originate from the tedious separation process of the products **3** from the byproduct, 4-toluenesulfonamide (4-MeC_6_H_4_SO_2_NH_2_) **5** produced by the hydrolysis of the unreacted imines **2** [15]. As expected, aromatic sulfonylimines with electron-withdrawing substituents **2bc**–**dc** acted nicely as electrophiles to furnish the desired adducts **3b**–**d** in high to excellent isolated yields where the complementary formation of the diastereomers was usually observed (Table 2, entries 3–8). Aromatic imines with electron-donating groups, **2fc** and **2gc**, as well as the heteroaromatic imine **2ec** sometimes required longer reaction times, but worked without any significant problems (Table 2, entries 9–14). As a general trend, LiHMDS tended to afford lower chemical yields the reason for which was not clear at present. Imines from aliphatic aldehydes **2hc** and **2ic** could be used in a similar manner but the steric effect due to the branched structure in the latter affected the reaction to some extent (Table 2, entries 15 and 16). As described above, facile isolation of **3** was sometimes hampered by contamination of the substrate **1a** and/or 4-toluenesulfonamide **5** which is noticed by the absence of isolated yields shown in brackets in Table 2.

**Figure 1:**
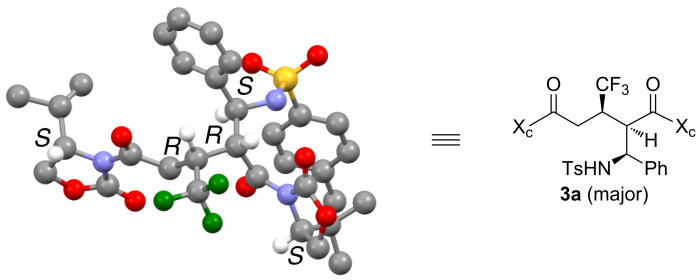
Crystallographic analysis of the major diastereomer of **3a** (some hydrogen atoms are omitted for clarity). Each color represents the following atoms: gray, carbon; white, hydrogen; blue, nitrogen; red, oxygen; green, fluorine; yellow, sulfur.

The configuration of the major diastereomer of **3a** (Table 2, entry 1) was unambiguously determined by X-ray crystallographic analysis (Figure 1) [16].

**Scheme 1:**
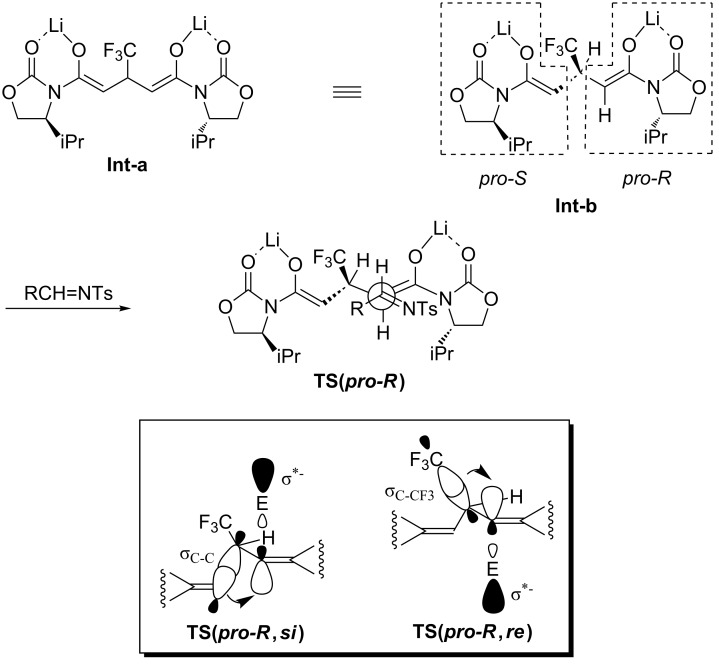
Explanation of the construction of the main stereoisomers.

Based on this information a possible reaction mechanism was formulated as depicted in Scheme 1. It is well-known that oxazolidinone-derived imides in general construct bidentate chelation when they are converted to the corresponding metal enolates [17]. The subsequent reactions occur so as to avoid unfavorable steric repulsive interaction with the oxazolidinone substituent, the iPr group in our case (**Int-a**). The selection of the two enantiotopic enolates should be explained on the basis of the Cieplak effect [18–19] for the enolate conformation with the allylic hydrogen possessing the same plane for making the steric bias minimum (**Int-b**) [20–21]. The most important argument for this effect is the stabilization of the forming electron-deficient σ^*≠^ orbital in the transition state by the electron donation from the neighboring orbital. In our case, possible electron donation is expected either by the σ_C-C_ in **TS(*pro-R*,*si***) or σ_C-CF3_ in **TS(*pro-R*,*re***). Because the former orbital is more electron-rich, imines as electrophiles should approach from the *si* face of the *pro-R* enolate (E: an appropriate electrophile in Scheme 1). The same orbital interaction would be operative when the electrophile came closer from the *re* face of the *pro-S* enolate which suffered from the existence of the sterically demanding iPr group. As a result, the major reaction pathway was considered to follow the transition state **TS(*pro-R*,*si***) where the *si* face of imines seemed to match favorably with a construction of the least sterically demanding conformation, leading to formation of the obtained diastereoisomer **3a**.

## Conclusion

As shown above, the oxazolidinone-installed imide from 3-(trifluoromethyl)glutaric acid **1a** was found to afford the corresponding adducts **3** in good to excellent chemical yields in a stereoselective fashion when its enolate was subjected to a solution containing tosylated imines **2** as electrophiles. Further work is going on in this laboratory to utilize the thus obtained adducts **3** and the results will be reported in due course.

## Supporting Information

File 1 Experimental procedures, characterization data, copies of ^1^H and ^13^C NMR spectra for the new compounds, and crystallographic analysis data are available.
