# Supplementary material for: Diastereoselective Mannich reactions of pseudo-C2-symmetric glutarimide with activated imines
Source: Beilstein J Org Chem. 2017 Nov 21;13:2473–7. doi: 10.3762/bjoc.13.244 (PMC5704755; doi:10.3762/bjoc.13.244)
Supplement: File 1 — Experimental procedures, characterization data, copies of 1H and 13C NMR spectra for the new compounds, and crystallographic analysis data are available. [file Beilstein_J_Org_Chem-13-2473-s001.pdf]

Supporting Information

for

Diastereoselective Mannich reactions of pseudo-C_2-symmetric glutarimide with activated imines

Tatsuya Ishikawa,[1] Tomoko Kawasaki-Takasuka,[1] Toshio Kubota,[2] and Takashi Yamazaki*[1]

Address: [1]Division of Applied Chemistry, Institute of Engineering, Tokyo University of Agriculture and Technology, 2-24-16 Nakamachi, Koganei 184-8588, Japan and [2]Department of Biomolecular Functional Engineering, Ibaraki University, Nakanarusawa 4-12-1, Hitachi 316-8511, Japan

Email: Takashi Yamazaki - tyamazak@cc.tuat.ac.jp

*Corresponding author

Experimental procedures, characterization data, copies of ^{1}H and ^{13}C NMR spectra for the new compounds, and crystallographic analysis data

Table of Contents

1. General information S2
2. Experimental procedures and characterization cata S3
General procedure for the reactions of the oxazolidinone-installed imide **1a** from 3-(trifluoromethyl)-glutaric acid with tosylated imines **2** S3
1,5-bis[(4*S*)-4-Isopropyl-2-oxo-1,3-oxazolidin-3-yl]-2-{phenyl(4-toluene-sulfonamido)methyl}-3-(trifluoromethyl)-pentane-1,5-dione (**3a**) S3
3. ^{1}H and ^{13}C NMR spectra for new compounds... S7
4. Crystallographic data for compound **3a** S13
5. References S15

1. General Information

^{1}H (300.40 MHz), ^{13}C (75.45 Hz), and ^{19}F (282.65 Hz) NMR spectra were recorded on a JEOL AL 300 spectrometer in $CDCl_3$ unless otherwise noted. Chemical shifts were recorded in parts per million (ppm) downfield from internal tetramethylsilane (Me_4Si: δ 0.00 ppm for ^{1}H and ^{13}C, respectively) or hexafluorobenzene (C_6F_6: δ −163.00 ppm for ^{19}F). Data were tabulated in the following order: number of protons, multiplicity (s, singlet; d, doublet; t, triplet; q, quartet; quint, quintet; sex, sextet; m, multiplet; b, broad peak), coupling constants in hertz. Infrared (IR) spectra were obtained on a JASCO A-302 spectrometer and reported in wave numbers (cm^{-1}). Elemental analyses were performed with a Perkin-Elmer Series II CHNS/O analyzer. JEOL JMS-700 was used for obtaining high resolution mass spectrometry data operated in the positive ionization mode.

Most of reactions where an organic solvent was employed were performed under argon atmosphere with magnetic stirring using flame-dried glassware. Anhydrous THF, Et_2O, and CH_2Cl_2 were purchased and used without further purification. Unless otherwise noted, materials were obtained from commercial suppliers and were used without further purification. Analytical thin-layer chromatography (TLC) was routinely used for monitoring reactions by generally using a mixture of hexane (Hex) and ethyl acetate (AcOEt) (v/v). Spherical neutral silica gel (63–210 μm or 40–50 μm) was employed for column chromatography and flush chromatography, respectively.

2. Experimental procedures and characterization data

General procedure for the reactions of the oxazolidinone-installed imide 1a from the 3-(trifluoromethyl)glutaric acid with tosylated imines 2.

1,5-bis[(4*S*)-4-Isopropyl-2-oxo-1,3-oxazolidin-3-yl]-2-{phenyl(4-toluenesulfon-amido)methyl}-3-(trifluoromethyl)pentane-1,5-dione (3a).

To a 30 mL two-necked flask containing THF (5 mL) and 0.08 mL (0.6 mmol) diisopropylamine under an argon atmosphere was added BuLi (0.36 mL, 0.58 mmol) at 0 °C and the mixture was stirred for 30 min at that temperature. After cooling to −80 °C, 1,5-bis[(4*S*)-4-isopropyl-2-oxo-1,3-oxazolidin-3-yl]-3-(trifluoromethyl)pentane-1,5-di-one [1] (**1a**, 0.0845 g, 0.200 mmol) was added to the LDA solution and stirred for 30 min at −80 °C where *N*-benzylidene-4-toluenesulfonamide [2](**2ac**, 0.1141 g 0.440 mmol) was added and the whole mixture was stirred for further 6 h at that temperature. Then, aqueous NH_4Cl was added, the mixture extracted three times with AcOEt, and the collected AcOEt layer was dried over anhydrous Na_2SO_4. Filtration and evaporation afforded a crude material which was chromatographed on silica gel using hexane/AcOEt 2:1 as an eluent to give 0.1026 g (0.15 mmol) of the desired 1,5-bis[(4*S*)-4-isopropyl-2-oxo-1,3-oxazolidin-3-yl]-2-{phenyl(4-toluenesulfonamido)-methyl}-3-(trifluoromethyl)pentane-1,5-dione (**3a**) as a 73:17:(10) mixture of diastereoisomers in 75% yield. The major stereoisomer was isolated by recrystallization (to the sample, dissolved in a small amount of AcOEt at 60 °C hexane was added until a clear solution was obtained. Cooling to room temperature afforded the desired main products) whose spectral data are given below.

Mp.: 186.5-187.0 °C, Rf=0.58 (hexane:AcOEt=1:1), $[\alpha]_D^{24}$ +33.0 (*c* 1.0, $CHCl_3$). ^1H NMR: δ 0.73 (3H, d, *J*=6.9 Hz), 0.87 (3H, d, *J*=7.2 Hz), 0.99 (3H, d, *J*=7.2 Hz), 1.01 (3H, d, *J*=6.6 Hz), 2.26–2.34 (1H, m), 2.30 (3H, s), 2.47–2.53 (1H, m), 2.86–3.00 (1H, m), 3.12 (1H, dd, *J*=18.6, 6.0 Hz), 3.60 (1H, dd, *J*=18.3, 6.0 Hz), 4.19–4.31 (4H, m), 4.34–4.38 (1H, m), 4.40–4.43 (1H, m), 4.77 (1H, t, *J*=9.9 Hz), 5.04 (1H, dd, *J*=9.0, 3.3 Hz), 5.76 (1H, d, *J*=10.2 Hz), 7.00 (2H, d, *J*=8.4 Hz), 7.10-7.14 (5H, m), 7.42 (2H, d, *J*=8.4 Hz). ^{13}C NMR: δ 14.5, 14.6, 18.0, 18.1, 21.3, 28.28, 28.33, 31.1, 38.1 (q, *J*=26.6 Hz), 43.7, 58.8, 59.4, 59.7, 63.5, 63.6, 126.9 (q, *J*=279.8 Hz), 126.9, 127.0, 128.1, 128.6, 129.1, 137.0, 137.2, 142.9, 154.0, 154.6, 169.6, 170.8. ^{19}F NMR: δ –71.96 (d, *J*=9.0 Hz), –70.30 (d, *J*=9.0 Hz; minor diastereomer). IR (KBr): ν 548, 559, 668, 705, 817, 1120, 1165, 1207, 1251, 1304, 1375, 1388, 1605, 1707, 1781, 2872, 2955, 3273, 3550, 3623 cm^{-1}. Anal. Calcd for $C_{32}H_{38}F_3N_3O_8S$: C, 56.38; H, 5.62; N, 6.16. Found: C, 56.16; H, 5.55; N, 6.16.

1,5-bis[(4*S*)-4-Isopropyl-2-oxo-1,3-oxazolidin-3-yl]-2-{(4-bromophenyl)(4-toluene-sulfonamido)methyl}-3-(trifluoromethyl)pentane-1,5-dione (3b)

91% yield as a 62:17:(21) mixture. mp.: 140.0–141.0 °C, Rf=0.58 (hexane: AcOEt=1:1). $[\alpha]_D^{26}$ +20.2 (*c* 0.89, $CHCl_3$). ^{1}H NMR: δ 0.72 (3H, d, *J*=6.9 Hz), 0.87 (3H, d, *J*=7.2 Hz), 1.00 (3H, d, *J*=6.9 Hz), 1.01 (3H, d, *J*=6.9 Hz), 2.21–2.32 (1H, m), 2.36 (3H, s), 2.46–2.57 (1H, m), 2.89–2.97 (1H, m), 3.05 (1H, dd, *J*=18.3, 5.4 Hz), 3.60 (1H, dd, *J*=18.3, 7.2 Hz), 4.18–4.29 (4H, m), 4.32–4.36 (1H, m), 4.41–4.46 (1H, m), 4.75 (1H, t, *J*=9.9 Hz), 5.00 (1H, dd, *J*=9.3, 3.3 Hz), 5.83 (1H, d, *J*=10.2 Hz), 7.01 (2H, d, *J*=8.4 Hz), 7.06 (2H, d, *J*=8.4 Hz), 7.24 (2H, d, *J*=8.4 Hz) 7.43 (2H, d, *J*=8.4 Hz). ^{13}C NMR: δ 14.5, 14.6, 18.0, 18.1, 21.1, 28.35, 28.44, 31.3, 38.1 (q, *J*=27.3 Hz), 43.5, 58.8, 59.0, 59.7, 63.6, 63.7, 122.3, 126.8 (q, *J*=279.7 Hz), 127.0, 128.9, 129.2, 131.7, 136.2, 137.1, 143.4, 154.0, 154.7, 169.7, 170.6. ^{19}F NMR: δ −72.03 (d, *J* = 9.0 Hz), −69.73 (d, *J* = 9.0 Hz; second major diastereomer), −70.70 (d, *J* = 9.3 Hz; minor diastereomer). IR (KBr): ν 545, 666, 716, 811, 855, 956, 1016, 1111, 1173, 1227, 1311, 1384, 1495, 1611, 1700, 1783, 2878, 2955, 3267, 3567 cm^{-1}. Anal. Calcd for $C_{32}H_{37}BrF_3N_3O_8S$: C, 50.53; H, 4.90; N, 5.52. Found: C, 50.35; H, 5.12; N, 5.07.

1,5-bis[(4*S*)-4-Isopropyl-2-oxo-1,3-oxazolidin-3-yl]-2-{(4-nitrophenyl)(4-toluene-sulfonamido)methyl}-3-(trifluoromethyl)pentane-1,5-dione (3c)

92% yield as a 62:13:(22) mixture. mp.: 140.5–143.0 °C, Rf=0.58 (hexane: AcOEt=1:1), $[\alpha]_D^{25}$ +0.72 (*c* 1.8, $CHCl_3$). ^{1}H NMR: δ 0.61 (3H, d, *J*=6.6 Hz), 0.84 (3H, d, *J*=6.9 Hz), 1.02 (3H, d, *J*=6.9 Hz), 1.02 (3H, d, *J*=6.9 Hz), 2.14–2.24 (1H, m), 2.32 (3H, s), 2.50–2.61 (1H, m), 2.92–3.00 (2H, m), 3.62 (1H, dd, *J*=18.3, 7.5 Hz), 4.17–4.33 (5H, m), 4.43–4.48 (1H, m), 4.91 (1H, t, *J*=9.0 Hz), 5.09 (1H, dd, *J*=8.7, 3.3 Hz), 6.01 (1H, d, *J*=9.6 Hz), 7.08 (2H, d, *J*=8.4 Hz), 7.41 (2H, d, *J*=8.7 Hz), 7.50 (2H, d, *J*=7.8 Hz) 8.01 (2H, d, *J*=8.7 Hz). ^{13}C NMR: δ 14.4, 14.6, 18.0, 18.1, 21.4, 28.3, 28.4, 31.5 (q, *J*=1.9 Hz), 38.1 (q, *J*=26.6 Hz), 43.4, 58.5, 59.1, 59.7, 63.6, 63.8, 123.7, 126.7 (q, *J*=279.8 Hz), 127.1, 128.4, 129.4, 143.7, 144.7, 147.5, 154.1, 154.5, 169.7, 170.3. ^{19}F NMR: δ −72.09 (d, *J*=9.3 Hz), −70.71 (d, *J*=9.3 Hz; second major diastereomer), −69.58 (d, *J*=9.3 Hz; third major diastereomer), −68.71 (d, *J*=9.3 Hz; minor diastereomer). IR (KBr): ν 537, 549, 561, 671, 703, 817, 1111, 1165, 1208, 1305, 1348, 1377, 1389, 1524, 1611, 1703, 1776, 2978, 3261, 3633 cm^{-1}. Anal. Calcd for $C_{32}H_{37}F_4N_3O_8S$: C, 54.93; H, 5.33; N, 6.01. Found: C, 53.10; H, 5.52; N, 7.72.

1,5-bis[(4*S*)-4-Isopropyl-2-oxo-1,3-oxazolidin-3-yl]-2-{(3-fluorophenyl)(4-toluene-sulfonamido)methyl}-3-(trifluoromethyl)pentane-1,5-dione (3d)

78% yield, a 81:6:(13) mixture. mp.: 160.8-161.5 °C, Rf=0.58 (hexane:AcOEt= 1:1), $[\alpha]_D^{24}$ +42.5 (*c* 5.7, $CHCl_3$). ^{1}H NMR: δ 0.72 (3H, d, *J*=6.9 Hz), 0.87 (3H, d, *J*=6.9 Hz), 0.99 (3H, d, *J*=6.9 Hz), 1.01 (3H, d, *J*=6.9 Hz), 2.27–2.32 (1H, m), 2.32 (3H, s), 2.50–2.53 (1H, m), 2.91–2.96 (1H, m), 3.05 (1H, dd, *J*=18.3, 5.4 Hz), 3.61 (1H, dd, *J*=18.6, 6.6 Hz), 4.19–4.29 (4H, m), 4.32–4.36 (1H, m), 4.41–4.45 (1H, m), 4.76 (1H, t, *J*=9.9 Hz), 5.03 (1H, dd, *J*=9.3, 3.3 Hz), 5.85 (1H, d, *J*=9.9 Hz), 6.74–6.77 (1H, m), 6.84 (1H, dt, *J*=8.4, 1.8 Hz), 6.97 (1H, d, *J*=8.1 Hz), 7.06 (2H, d, *J*=8.1 Hz), 7.14 (1H, dt, *J*=8.1, 6.0 Hz), 7.46 (2H, d, *J*=8.1 Hz). ^{13}C NMR: δ 14.3, 14.5, 17.75, 17.78, 17.83, 17.86, 21.08, 21.11, 28.1, 31.0, 37.9 (q, *J*=26.7 Hz), 43.4, 58.8, 59.5, 63.5, 114.3 (d, *J*=22.3 Hz), 114.8 (d, *J* = 21.1 Hz), 122.4, 126.7 (q, *J*=279.8 Hz), 126.9, 129.0, 130.2 (d, *J*=8.1 Hz), 136.9, 139.6 (d, *J*=6.9 Hz), 143.0 (d, *J*=1.2 Hz), 154.0, 154.3, 162.4 (d, *J*=246.9 Hz), 169.4, 170.5. ^{19}F NMR: δ −113.72 (1F, m), −114.00 (1F, m; second major diastereomer), −112.57 (1F, m; third major diastereomer and the peak for the minor diastereomer was not found out possibly due to overlap), −71.95 (3F, d, *J*=9.0 Hz), −67.73 (3F, d, *J*=9.0 Hz; second major diastereomer), −69.58 (3F, d, *J*=9.0 Hz; third major diastereomer), −70.57 (3F, d, *J*=9.4 Hz; minor diastereomer). IR (KBr): ν 553, 666, 671, 703, 812, 1093, 1120, 1164, 1205, 1245, 1304, 1342, 1389, 1488, 1707, 1781, 2361, 2967, 3277, 3639 cm^{-1}. Anal. Calcd for $C_{32}H_{37}F_4N_3O_8S$: C, 54.93; H, 5.33; N, 6.01. Found: C, 54.58; H, 5.45; N, 5.97.

1,5-bis[(4*S*)-4-Isopropyl-2-oxo-1,3-oxazolidin-3-yl]-2-{2-furyl(4-toluenesulfon-amido)methyl}-3-(trifluoromethyl)-pentane-1,5-dione (inseparable diastereomer mixture) (3e)

55% yield, a mixture of 51:22:(27). Rf=0.58 (hexane:AcOEt=1:1). ^{1}H NMR: δ 0.91 (3H, d, *J*=6.6 Hz), 0.92 (3H, d, *J*=6.9 Hz), 0.98 (3H, d, *J*=8.1 Hz), 1.00 (3H, d, *J*=7.9 Hz), 2.34 (3H, s), 2.40–2.51 (1H, m), 2.86–2.95 (1H, m), 3.17 (1H, dd, *J*=18.3, 6.3 Hz), 3.56 (1H, dd, *J*=18.6, 6.3 Hz), 4.24–4.33 (5H, m), 4.41–4.45 (2H, m), 4.41–4.45 (1H, m), 4.87 (1H, t, *J*=10.2 Hz), 5.09 (1H, dd, *J*=10.2, 2.4 Hz), 5.59 (1H, d, *J*=10.5 Hz), 6.00–6.05 (2H, m), 7.10–7.14 (2H, m), 7.34 (1H, t, *J*=9.0 Hz), 7.49 (2H, d, *J*=8.4 Hz). ^{13}C NMR: δ 14.5, 14.7, 17.99, 18.02, 21.4, 28.2, 31.10, 31.12, 38.2 (q, *J*=26.6 Hz), 41.7, 53.5, 58.7, 59.6, 63.47, 63.55, 109.8, 110.0, 126.7 (q, *J*=279.7 Hz), 126.9, 129.2, 137.0, 142.8, 143.0, 148.7, 153.9, 154.3, 169.8, 170.3. ^{19}F NMR: δ −71.85 (d, *J*=9.0 Hz), −70.59 (d, *J*=9.3 Hz; second major diastereomer), −69.88 (d, *J*=9.0 Hz; third major diastereomer), −67.43 (d, *J*=9.0 Hz; minor diastereomer). IR (KBr): ν 495, 555, 666,

700, 744, 811, 933, 1066, 1117, 1167, 1217, 1306, 1394, 1589, 1716, 1778, 2883, 2961, 3267, 3545 cm^{-1}. HRMS-FAB (*m*/*z*) : $[M+H]^+$ calcd. for $C_{30}H_{36}F_3N_3O_9S$, 672.2203; found, 672.2197.

1,5-bis[(4*S*)-4-Isopropyl-2-oxo-1,3-oxazolidin-3-yl]-2-{(4-methylphenyl)(4-toluene-sulfonamido)methyl}-3-(trifluoromethyl)pentane-1,5-dione (3f)

66% yield, a 51:16:(33) mixture. mp.: 129.0-131.0 °C, Rf=0.50 (hexane: AcOEt=1:1), $[\alpha]_D^{21}$ +67.7 (*c* 0.56, $CHCl_3$). ^1H NMR: δ 0.74 (3H, d, *J*=6.9 Hz), 0.88 (3H, d, *J*=6.9 Hz), 0.98 (3H, d, *J*=6.9 Hz), 1.00 (3H, d, *J*=6.6 Hz), 2.24 (3H, s), 2.29–2.34 (1H, m), 2.31 (3H, s), 2.43–2.54 (1H, m), 2.88–3.02 (1H, m), 3.14 (1H, dd, *J*=18.6, 6.6 Hz), 3.59 (1H, dd, *J*=18.3, 6.0 Hz), 4.19–4.31 (4H, m), 4.35–4.37 (1H, m), 4.39–4.43 (1H, m), 4.72 (1H, t, *J*=9.9 Hz), 5.00 (1H, dd, *J*=9.9, 3.3 Hz), 5.71 (1H, d, *J*=10.2 Hz), 6.89–7.02 (6H, m), 7.41 (2H, d, *J*=8.1 Hz). ^{13}C NMR: δ 14.5, 14.7, 18.0, 18.1, 21.0, 21.3, 28.3, 28.4, 31.0, 38.2 (q, *J*=26.7 Hz), 43.7, 58.9, 59.2, 59.7, 63.5, 63.6, 126.8, 126.9 (q, *J*=280.4 Hz), 127.1, 129.0, 129.2, 134.0, 137.3, 138.0, 142.8, 153.9, 154.7, 169.7, 170.9. ^{19}F NMR: δ −71.95 (d, *J* = 9.0 Hz), −69.60 (d, *J* = 9.0 Hz; second major diastereomer), −70.56 (d, *J* = 9.3 Hz; third major diastereomer), −73.14 (d, *J* = 9.0 Hz; minor diastereomer). IR (KBr): ν 539, 569, 665, 1094, 1162, 1208, 1256, 1305, 1332, 1366, 1393, 1405, 1611, 1686, 1697, 1760, 1797, 2972, 3100, 3577 cm^{-1}. HRMS-FAB (*m*/*z*) : $[M+2H]^+$ calcd. for $C_{33}H_{42}F_3N_3O_8S$, 697.2645; found, 697.2675.

3. ^{1}H and ^{13}C NMR spectra

1,5-bis[(4*S*)-4-Isopropyl-2-oxo-1,3-oxazolidin-3-yl]-2-{phenyl(4-toluenesulfonamido)methyl}-3-(trifluoromethyl)pentane-1,5-dione (**3a**)

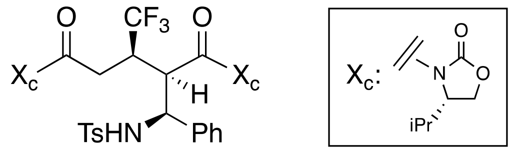

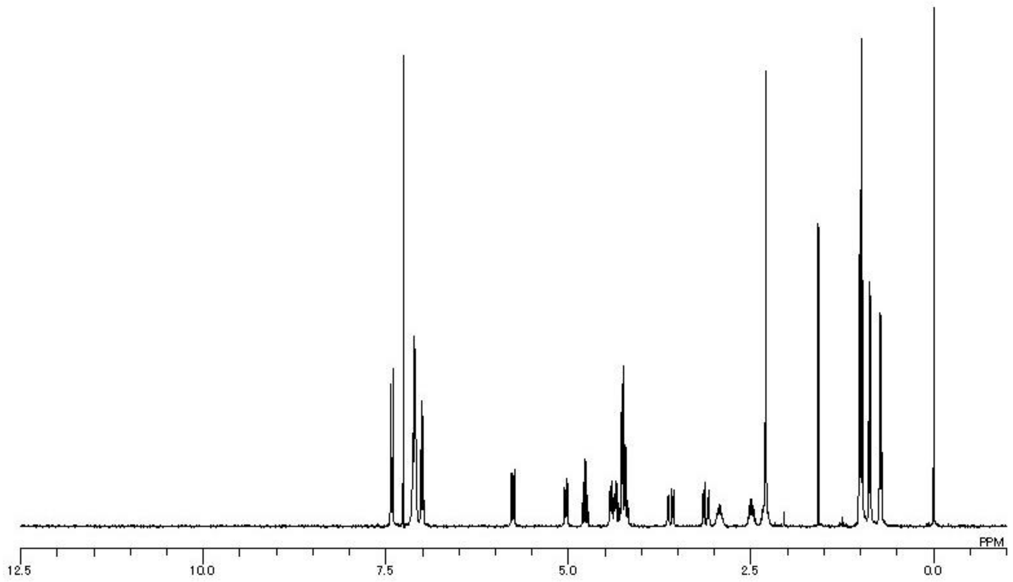

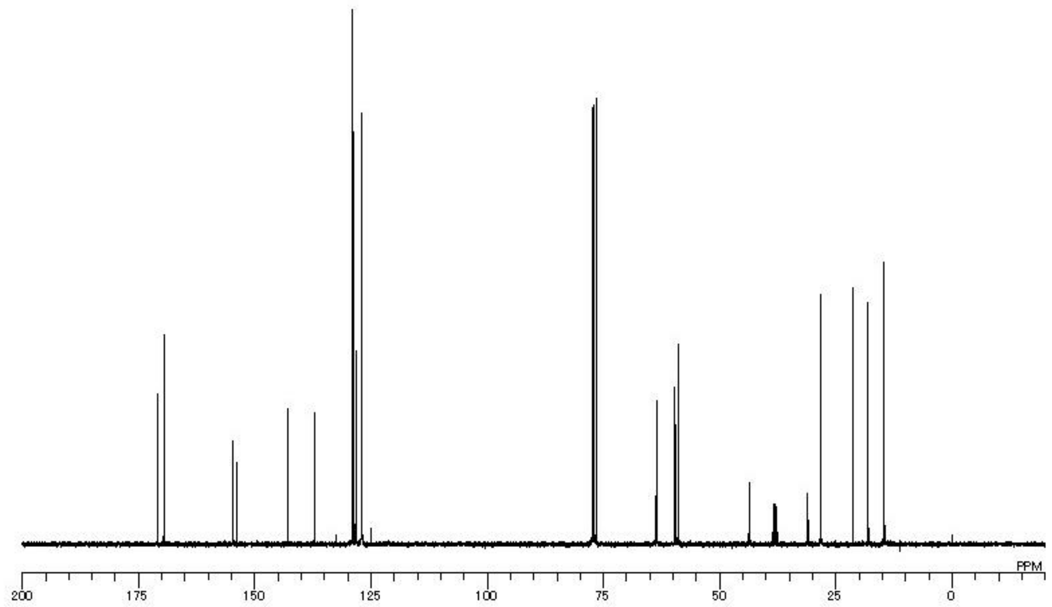

1,5-bis[(4*S*)-4-Isopropyl-2-oxo-1,3-oxazolidin-3-yl]-2-{(4-bromophenyl)(4-toluenesulfonamido)-methyl}-3-(trifluoromethyl)pentane-1,5-dione (**3b**)

(Stereochemistry was assumed on the basis of the one of **3a**)

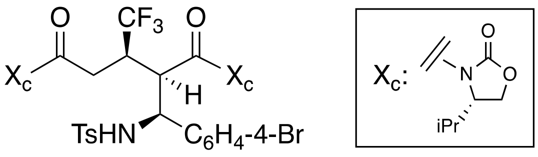

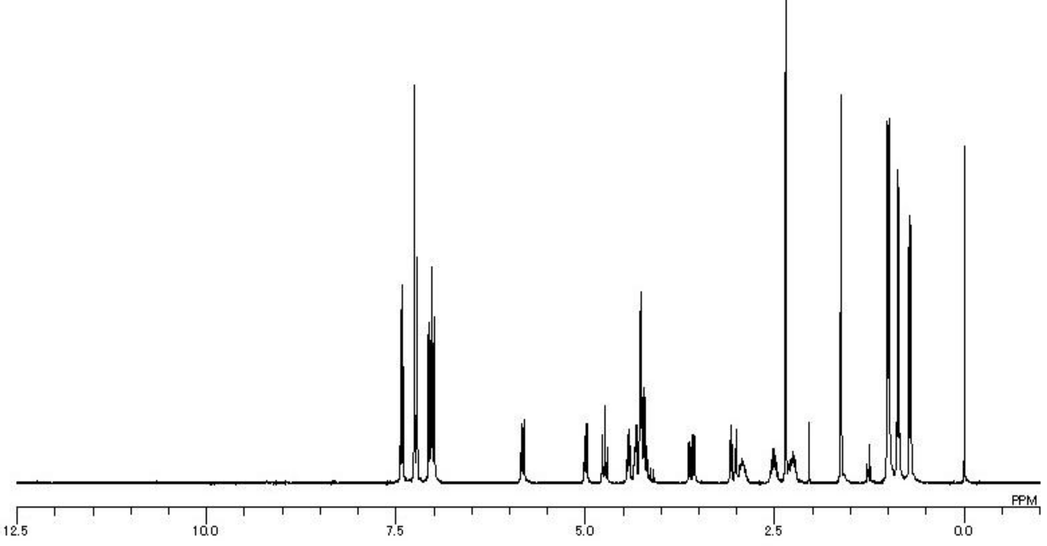

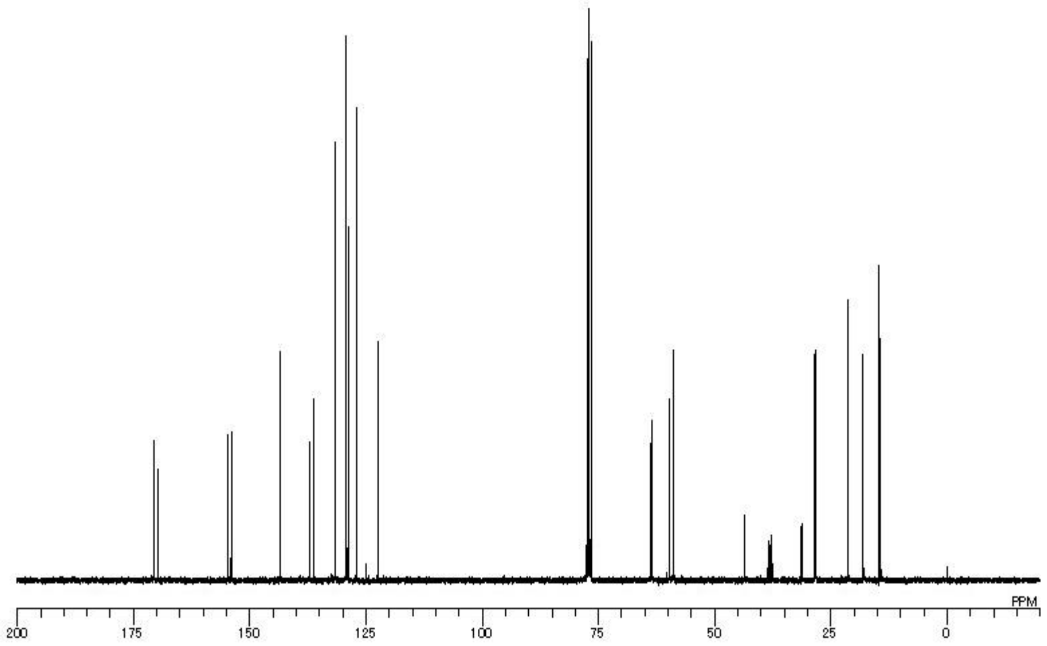

1,5-bis[(4*S*)-4-Isopropyl-2-oxo-1,3-oxazolidin-3-yl]-2-{(4-nitrophenyl)(4-toluenesulfonamido)-methyl}-3-(trifluoromethyl)pentane-1,5-dione (**3c**)
(Stereochemistry was assumed on the basis of the one of **3a**)

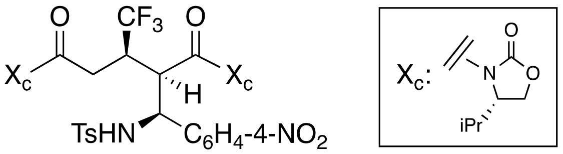

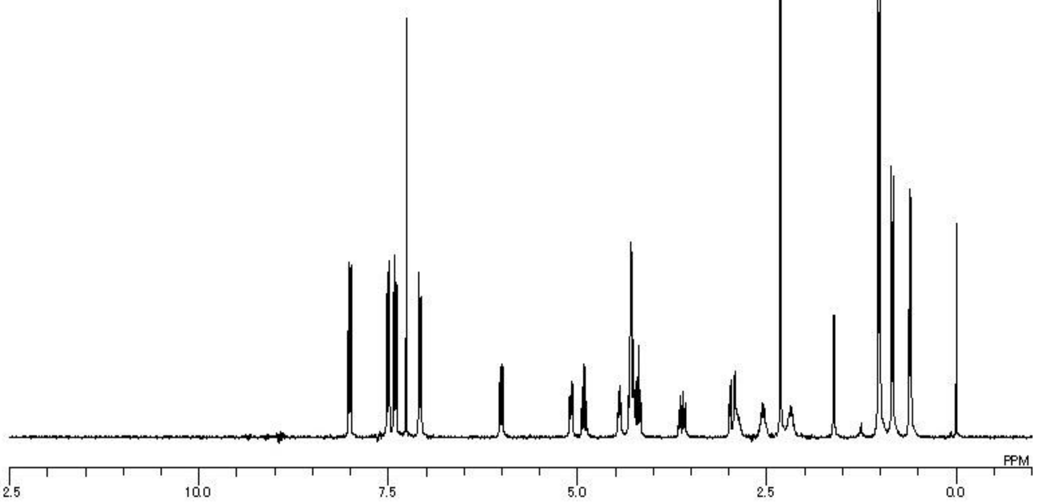

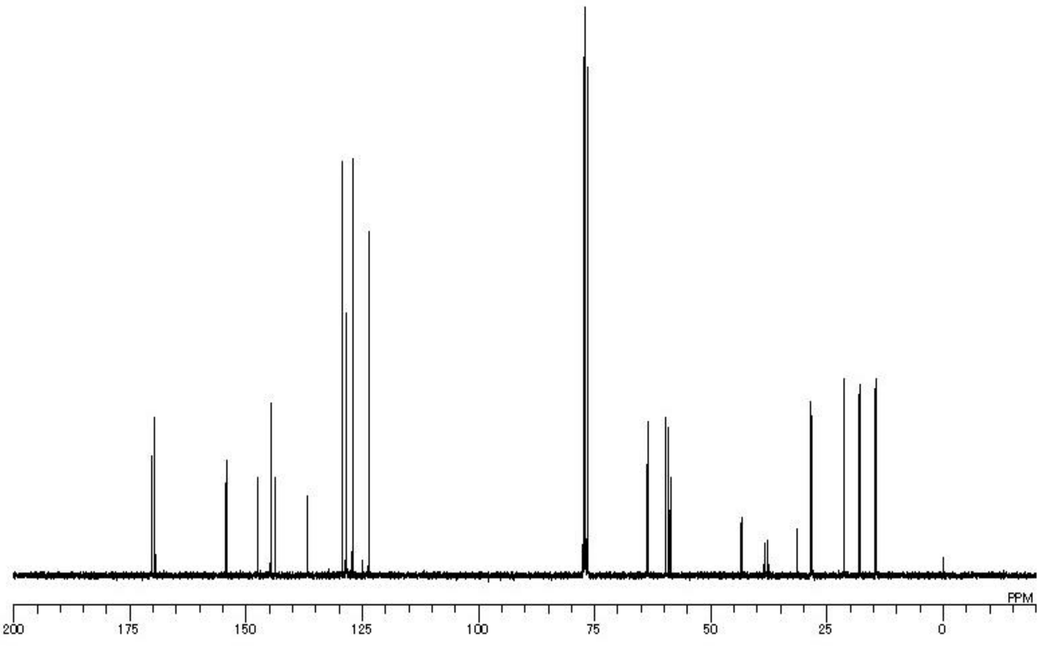

1,5-bis[(4*S*)-4-Isopropyl-2-oxo-1,3-oxazolidin-3-yl]-2-{(3-fluorophenyl)(4-toluenesulfonamido)-methyl}-3-(trifluoromethyl)pentane-1,5-dione (**3d**)

(Stereochemistry was assumed on the basis of the one of **3a**)

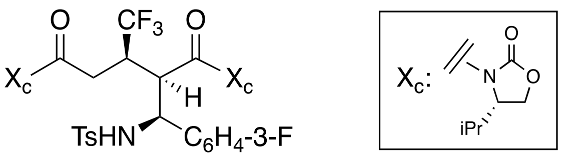

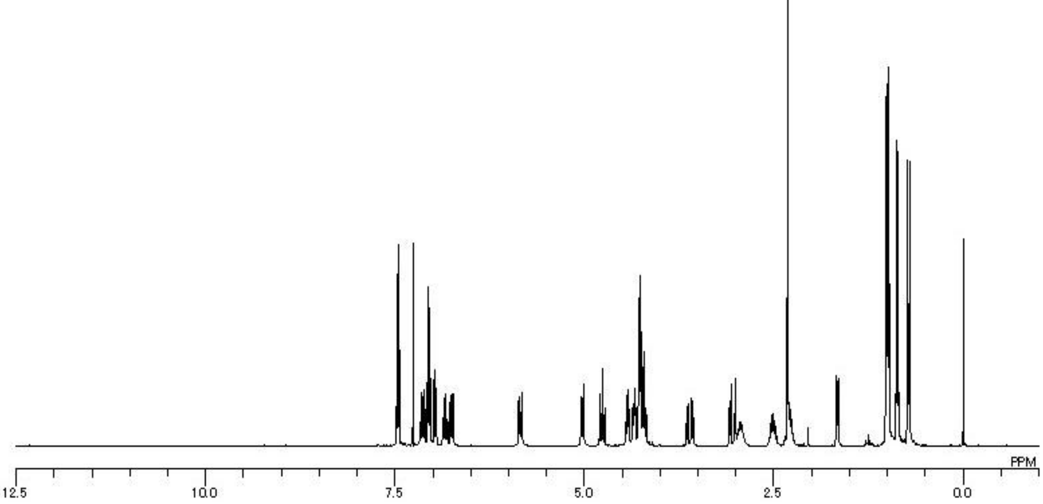

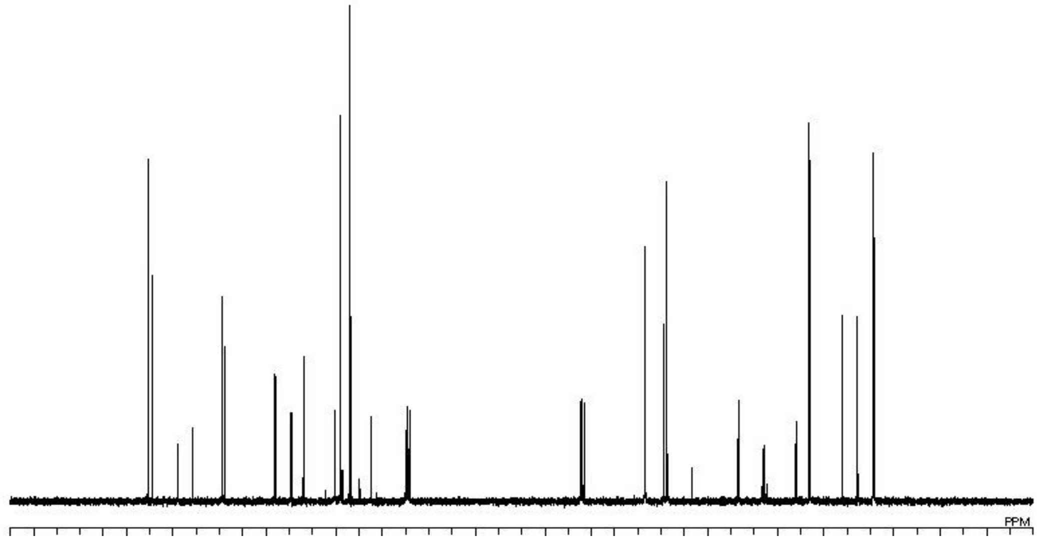

1,5-bis[(4*S*)-4-Isopropyl-2-oxo-1,3-oxazolidin-3-yl]-2-{2-furyl(4-toluenesulfonamido)methyl}-3-(trifluoromethyl)-pentane-1,5-dione (inseparable diastereomer mixture) (**3e**)

(Stereochemistry was assumed on the basis of the one of **3a**)

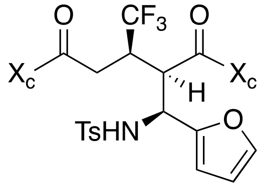

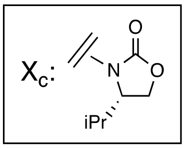

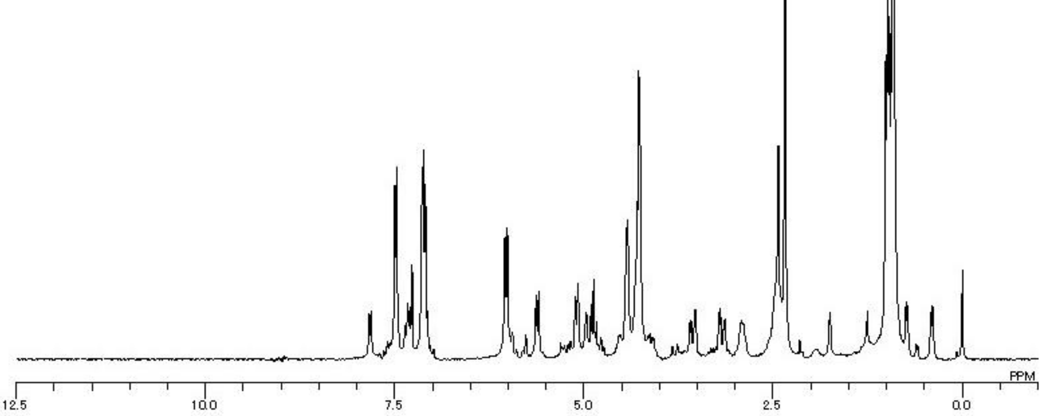

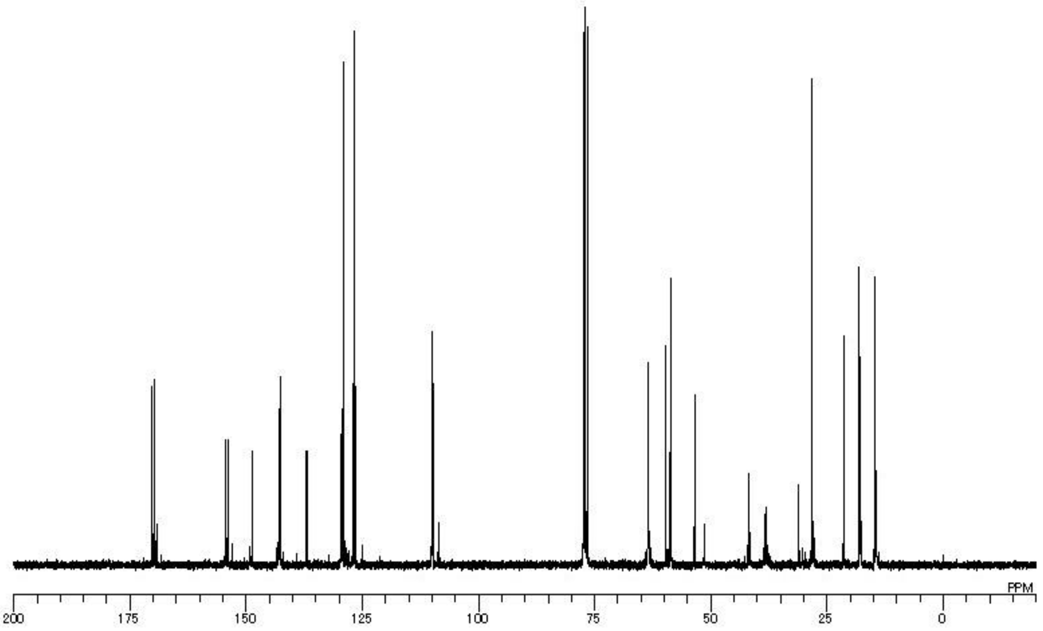

1,5-bis[(4*S*)-4-Isopropyl-2-oxo-1,3-oxazolidin-3-yl]-2-{(4-methylphenyl)(4-toluenesulfonamido)-methyl}-3-(trifluoromethyl)pentane-1,5-dione (**3f**)

(Stereochemistry was assumed on the basis of the one of **3a**)

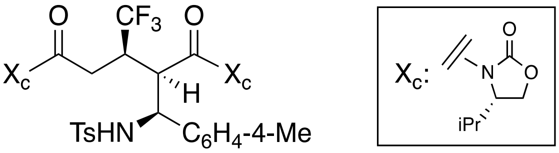

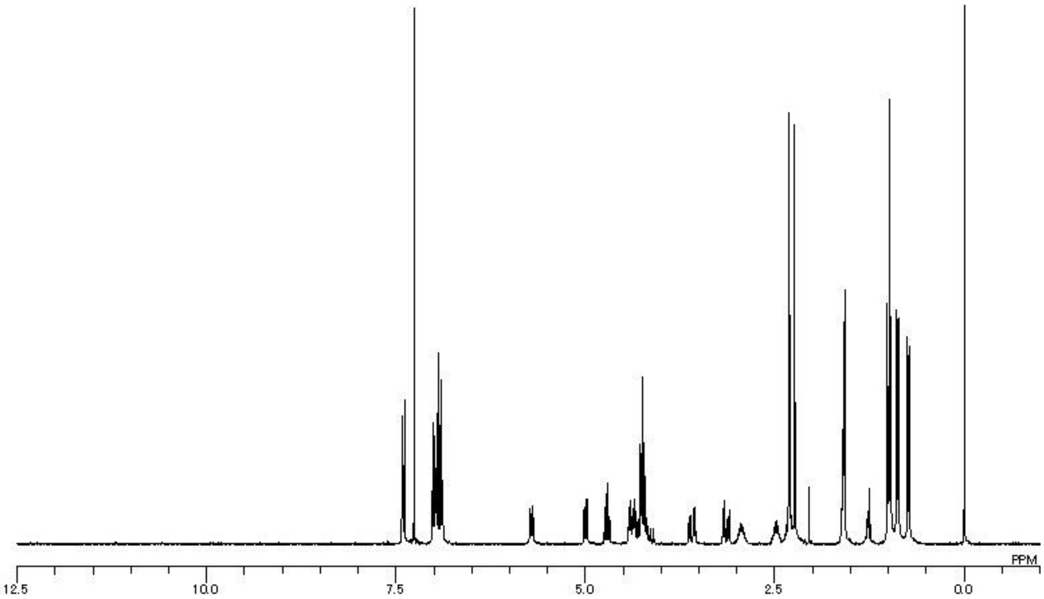

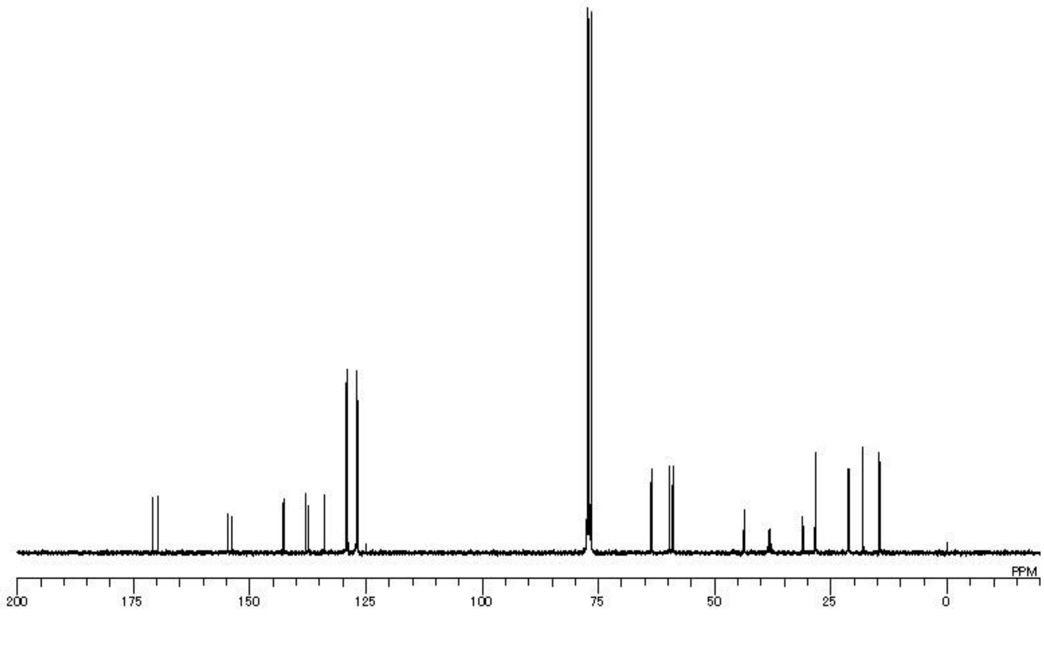

4. Crystallographic data of the major isomer of compound 3a

Deposition number	CCDC 1575731	
Empirical formula	C32 H38 F3 N3 O8 S	
Formula weight	681.71	
Temperature	173(2) K	
Wavelength	0.71073 Å	
Crystal system	Orthorhombic	
Space group	$P2_12_12_1$	
Unit cell dimensions	a = 11.5242(5) Å	α = 90°
	b = 15.0568(6) Å	β = 90°
	c = 19.4473(8) Å	γ = 90°
Volume	3374.4(2) $Å^3$	
Z	4	
Density (calculated)	1.342 Mg/m^3	
Absorption coefficient	0.166 mm^{-1}	
F(000)	1432	
Crystal size	0.230 x 0.210 x 0.170 mm^3	
Theta range for data collection	1.710 to 25.495°	
Index ranges	–13<=h<=13, –18<=k<=18, –23<=l<=23	
Reflections collected	30358	
Independent reflections	6261 [R(int) = 0.0325]	
Completeness to theta = 25.242°	99.7%	
Absorption correction	Semi-empirical from equivalents	
Max. and min. transmission	1.00000 and 0.87379	
Refinement method	Full-matrix least-squares on F^2	
Data / restraints / parameters	6261 / 1 / 429	
Goodness-of-fit on F^2	1.041	
Final R indices [I>2sigma(I)]	R1 = 0.0415, wR2 = 0.1011	
R indices (all data)	R1 = 0.0531, wR2 = 0.1104	
Absolute structure parameter	–0.01(2)	
Extinction coefficient	n/a	
Largest diff. peak and hole	0.231 and –0.234 e.$Å^{-3}$	

Table S1. Atomic coordinates (x10^4) and equivalent isotropic displacement parameters ($Å^2$x10^3). U(eq) is defined as one third of the trace of the orthogonalized Uij tensor.

	x	y	z	U(eq)
O(1)	5347(2)	3420(2)	3319(1)	54(1)
O(2)	5690(4)	6178(2)	3589(2)	90(1)
O(3)	5873(4)	5743(2)	4677(1)	98(1)
O(4)	1558(2)	4058(2)	1842(2)	73(1)
O(5)	2792(3)	1691(2)	2663(2)	80(1)
O(6)	1369(3)	1130(2)	2026(2)	87(1)
O(7)	8449(2)	4849(2)	1629(2)	79(1)
O(8)	7004(3)	3754(2)	1214(1)	85(1)
C(1)	4747(3)	5034(2)	1481(2)	45(1)
C(2)	4756(3)	5949(2)	1439(2)	56(1)
C(3)	4235(4)	6377(3)	890(2)	70(1)
C(4)	3709(4)	5897(3)	380(2)	78(1)
C(5)	3705(5)	4999(3)	410(2)	83(1)
C(6)	4218(4)	4557(3)	960(2)	66(1)
C(7)	5298(3)	4549(2)	2081(2)	41(1)
C(8)	4669(3)	4739(2)	2765(2)	41(1)
C(9)	5273(3)	4223(2)	3336(1)	42(1)
C(10)	5732(4)	5595(3)	4003(2)	71(1)
C(11)	5754(6)	4937(3)	5058(2)	98(2)
C(12)	5998(4)	4199(3)	4539(2)	63(1)
C(13)	7220(4)	3846(3)	4530(2)	75(1)
C(14)	8155(5)	4556(4)	4447(3)	104(2)
C(15)	7434(6)	3290(4)	5187(3)	111(2)
N(1)	6531(2)	4777(2)	2172(1)	46(1)
C(16)	3358(3)	4502(2)	2727(2)	46(1)
C(17)	2734(3)	4814(3)	3364(2)	67(1)
C(18)	3066(3)	3527(2)	2582(2)	51(1)
C(19)	2045(3)	3436(2)	2097(2)	52(1)
C(20)	2034(4)	1797(2)	2249(2)	61(1)

C(21)	448(4)	1464(3)	1607(3)	86(1)
C(22)	730(3)	2418(3)	1443(2)	65(1)
C(26)	8057(3)	3443(2)	2355(2)	51(1)
C(28)	7965(5)	2009(3)	2865(3)	91(2)
C(29)	8925(4)	2178(3)	3265(3)	79(1)
C(30)	9438(4)	2997(3)	3197(2)	82(1)
C(31)	9015(3)	3639(3)	2747(2)	65(1)
C(32)	9394(6)	1476(4)	3751(4)	131(2)
S(1)	7528(1)	4231(1)	1764(1)	56(1)
F(1)	3076(2)	4384(2)	3936(1)	93(1)
F(2)	1584(2)	4689(2)	3323(2)	94(1)
F(3)	2895(3)	5675(2)	3496(2)	96(1)
N(2)	5682(3)	4681(2)	3901(1)	51(1)
N(3)	1679(2)	2574(2)	1939(2)	54(1)
C(33)	1043(6)	2563(6)	711(3)	143(3)
C(34)	2064(7)	2527(10)	442(3)	226(6)
C(35)	–9(7)	2338(6)	251(4)	157(3)
C(36)	7530(4)	2625(3)	2409(2)	73(1)

5. References

1. Watanabe, Y.; Yamazaki, T.; Kubota, T. *Org. Lett.* **2010**, *12*, 268–271.
2. Novacek, J.; Roiser, L.; Zielke, K.; Robiette, R.; Waser, M. *Chem. Eur. J.* **2016**, *22*, 11422–11428.
